# Preparedness of non-hospital health centers to deal with life-threatening emergencies: a ‎qualitative study of healthcare providers’ and experts’ perspectives 

**Published:** 2019-08

**Authors:** Mehrdad Amir Behghadami, Ali Janati, Homayoun Sadeghi-Bazargani, Masoumeh Gholizadeh, Farzad Rahmani

**Affiliations:** ^*a*^Iranian Center of Excellence in Health Management (IceHM), Department of Health Service Management, School of Health Services Management and Medical Informatics, Tabriz University of Medical Sciences, Tabriz, Iran.; ^*b*^Department of Statistics and Epidemiology, Faculty of Health, Tabriz University of Medical Sciences, Tabriz, Iran.; ^*c*^Emergency Medicine Department, Sina Medical Research and Training Hospital, Tabriz University of Medical Sciences, Tabriz, Iran.

## Abstract

**Background:**

The life-threatening emergency (LTE) in urban and rural areas is an important challenge, which ‎can affect pre-hospital mortality rates. Therefore, non-hospital centers must be prepared to deal ‎with such emergency cases that may occur in the geographic area of activity of these centers. ‎The aim of this study was to identify prevalent domains related to the preparedness of non-‎hospital centers to deal with the LTE using of healthcare providers’ and experts’ perspectives. ‎

**Methods:**

A qualitative exploratory study was applied using Semi-Structured Interviews (SSIs) and Focus ‎Group Discussions (FGDs). Prior to beginning on data collection, the study and its aims were ‎explained to participants and their informed consents were obtained. Then SSIs and FGDs were ‎conducted by two trained researchers using an interview guide, which was developed through ‎literature review and consulting experts. In total, 12 SSIs were carried out with 4 clinical ‎officers, 2 nurses and 6 physicians at different non-hospital centers in Tabriz. In addition, 2 ‎FGDs were conducted with specialists in emergency medicine, primary health care, and ‎executives of health centers, with over 5 years of work experience. Purposive sampling method ‎was used as tool to select participants who have sufficient knowledge in our research scope. All ‎SSIs and FGDs were transcribed and immediately recorded word by word. Framework Analysis ‎was employed to manually analyze the interview transcripts from all the SSIs and FGDs. ‎

**Results:**

The interview transcripts analysis resulted in the emergence of 3 domains and 11 sub-domains, ‎categorized according to Donabedian’s triple model. 3 sub-domains were related to inputs, ‎including fundamental infrastructures, support facilities, and human resources. 6 sub-domains ‎were related to processes, including clinical interventions, maintenance of equipment, medicine ‎storage capability, educating and training, infection control, and quality control. Finally, 1 sub-‎domain was related to outcome, which was also patient’s satisfaction. ‎

**Conclusions:**

Existence of an efficient and effective assessment system can play a pivotal role in identifying ‎the capabilities of non-hospital centers. Therefore, the results of this study can assist health ‎managers to assess the preparedness of these centers.‎

**Keywords:**

Non-hospital Health centers, Preparedness, Assess, Life-threatening emergency

